# Valve type and post-dilation impact on transprosthetic gradients in patients undergoing transcatheter aortic valve-in-valve procedure

**DOI:** 10.1093/ehjimp/qyaf048

**Published:** 2025-05-14

**Authors:** Manuela Muratori, Laura Fusini, Gloria Tamborini, Paola Gripari, Sarah Ghulam Ali, Valentina Mantegazza, Anna Garlaschè, Francesco Doni, Andrea Baggiano, Francesco Cannata, Alberico Del Torto, Fabio Fazzari, Antonio Frappampina, Daniele Junod, Riccardo Maragna, Saima Mushtaq, Luigi Tassetti, Alessandra Volpe, Stefano Galli, Franco Fabbiocchi, Marco Gennari, Marco Agrifoglio, Antonio L Bartorelli, Federico De Marco, Mauro Pepi, Gianluca Pontone

**Affiliations:** Perioperative Cardiology and Cardiovascular Imaging Department, Centro Cardiologico Monzino IRCCS, via Parea 4, 20138 Milan, Italy; Perioperative Cardiology and Cardiovascular Imaging Department, Centro Cardiologico Monzino IRCCS, via Parea 4, 20138 Milan, Italy; Department of Electronics, Information and Biomedical Engineering, Politecnico di Milano, via Ponzio 34/5, 20133 Milan, Italy; Perioperative Cardiology and Cardiovascular Imaging Department, Centro Cardiologico Monzino IRCCS, via Parea 4, 20138 Milan, Italy; Perioperative Cardiology and Cardiovascular Imaging Department, Centro Cardiologico Monzino IRCCS, via Parea 4, 20138 Milan, Italy; Perioperative Cardiology and Cardiovascular Imaging Department, Centro Cardiologico Monzino IRCCS, via Parea 4, 20138 Milan, Italy; Perioperative Cardiology and Cardiovascular Imaging Department, Centro Cardiologico Monzino IRCCS, via Parea 4, 20138 Milan, Italy; Department of Clinical Sciences and Community Health, Cardiovascular Section, University of Milan, via della Commenda 19, 20122 Milan, Italy; Perioperative Cardiology and Cardiovascular Imaging Department, Centro Cardiologico Monzino IRCCS, via Parea 4, 20138 Milan, Italy; Perioperative Cardiology and Cardiovascular Imaging Department, Centro Cardiologico Monzino IRCCS, via Parea 4, 20138 Milan, Italy; Department of Clinical Sciences and Community Health, Cardiovascular Section, University of Milan, via della Commenda 19, 20122 Milan, Italy; Perioperative Cardiology and Cardiovascular Imaging Department, Centro Cardiologico Monzino IRCCS, via Parea 4, 20138 Milan, Italy; Department of Clinical Sciences and Community Health, Cardiovascular Section, University of Milan, via della Commenda 19, 20122 Milan, Italy; Perioperative Cardiology and Cardiovascular Imaging Department, Centro Cardiologico Monzino IRCCS, via Parea 4, 20138 Milan, Italy; Perioperative Cardiology and Cardiovascular Imaging Department, Centro Cardiologico Monzino IRCCS, via Parea 4, 20138 Milan, Italy; Perioperative Cardiology and Cardiovascular Imaging Department, Centro Cardiologico Monzino IRCCS, via Parea 4, 20138 Milan, Italy; Perioperative Cardiology and Cardiovascular Imaging Department, Centro Cardiologico Monzino IRCCS, via Parea 4, 20138 Milan, Italy; Perioperative Cardiology and Cardiovascular Imaging Department, Centro Cardiologico Monzino IRCCS, via Parea 4, 20138 Milan, Italy; Perioperative Cardiology and Cardiovascular Imaging Department, Centro Cardiologico Monzino IRCCS, via Parea 4, 20138 Milan, Italy; Perioperative Cardiology and Cardiovascular Imaging Department, Centro Cardiologico Monzino IRCCS, via Parea 4, 20138 Milan, Italy; Perioperative Cardiology and Cardiovascular Imaging Department, Centro Cardiologico Monzino IRCCS, via Parea 4, 20138 Milan, Italy; Perioperative Cardiology and Cardiovascular Imaging Department, Centro Cardiologico Monzino IRCCS, via Parea 4, 20138 Milan, Italy; Department of Interventional Cardiology, Centro Cardiologico Monzino IRCCS, via Parea 4, 20138 Milan, Italy; Interventional Cardiology Unit, IRCCS Ospedale Galeazzi Sant'Ambrogio, via Belgioioso 173, 20157 Milan, Italy; Department of Cardiovascular Surgery, Centro Cardiologico Monzino IRCCS, via Parea 4, 20138 Milan, Italy; Department of Cardiovascular Surgery, Centro Cardiologico Monzino IRCCS, via Parea 4, 20138 Milan, Italy; Department of Biomedical, Surgical and Dental Sciences, University of Milan, via della Commenda 10, 20122 Milan, Italy; Interventional Cardiology Unit, IRCCS Ospedale Galeazzi Sant'Ambrogio, via Belgioioso 173, 20157 Milan, Italy; Department of Interventional Cardiology, Centro Cardiologico Monzino IRCCS, via Parea 4, 20138 Milan, Italy; Perioperative Cardiology and Cardiovascular Imaging Department, Centro Cardiologico Monzino IRCCS, via Parea 4, 20138 Milan, Italy; Perioperative Cardiology and Cardiovascular Imaging Department, Centro Cardiologico Monzino IRCCS, via Parea 4, 20138 Milan, Italy; Department of Biomedical, Surgical and Dental Sciences, University of Milan, via della Commenda 10, 20122 Milan, Italy

**Keywords:** structural valve degeneration, aortic bioprosthesis, transcatheter aortic valve replacement, valve in valve, echocardiography

## Abstract

**Aims:**

Valve-in-Valve transcatheter aortic valve replacement (ViV-TAVR) is an appealing treatment option for patients with degenerated aortic bioprosthetic valves. However, higher post-procedural transprosthetic gradients are more common after ViV-TAVR than after TAVR for native aortic valve stenosis. We sought to evaluate the impact of type of implanted valve and balloon post-dilation on echocardiographic results and mortality in ViV-TAVR patients.

**Methods and results:**

One hundred and eleven consecutive patients were enrolled. A balloon-expandable valve, a self-expandable valve without balloon post-dilation, and a self-expandable valve with balloon post-dilation were performed in 35 (Group 1), 39 (Group 2), and 37 (Group 3) patients, respectively. All patients underwent comprehensive transthoracic echocardiography at baseline, discharge, and 6–12 months follow-up. Successful ViV-TAVR was performed in 110 patients (99%). Baseline transprosthetic gradients, left ventricular volumes, ejection fraction, and pulmonary artery systolic pressure were similar among groups. All groups experienced a significant reduction in post-procedural gradients at discharge and during the 6–12 months follow-up compared with baseline. At discharge, the lowest mean gradient was observed in Group 3 (12 ± 7 mmHg) compared with both Group 1 (20 ± 9 mmHg) and Group 2 (17 ± 8 mmHg, *P* = 0.001). This result was confirmed at 6–12 months follow-up (*P* = 0.012). Similar 5-year all-cause mortality was observed among groups (34%, 36%, 14%, respectively, *P* = 0.056).

**Conclusion:**

In patients with failed surgical aortic prosthesis, ViV-TAVR is an effective treatment option associated with sustained improved haemodynamics regardless of transcatheter valve type and use of balloon post-dilation. However, self-expandable valves with balloon post-dilation showed lower transprosthetic gradients.

## Graphical Abstract

**Graphical Abstract:**
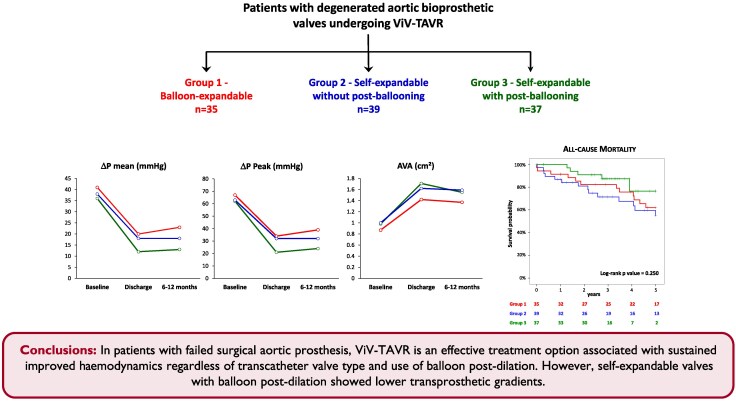

## Introduction

Biological valves are increasingly implanted in patients requiring aortic valve replacement, rather than mechanical valves.^1^ Bioprosthetic valves do not require anticoagulant therapy and have excellent haemodynamic properties. However, their durability is limited because of structural valve degeneration (SVD). Over time, SVD results in prosthesis failure and haemodynamic dysfunction manifested as stenosis, regurgitation, or a combination of both requiring the need for intervention in most cases.^2,3^ The conventional treatment for dysfunctional valve prosthesis has been redo surgery for many years. However, patients with SVD are often elderly and have several comorbidities, which frequently makes them unsuitable candidates for reoperation.^4,5^ Valve-in-Valve transcatheter aortic valve replacement (ViV-TAVR) has been identified as a feasible, less-invasive treatment option for patients with degenerated surgically implanted bioprostheses. Currently, European and US guidelines recommend this approach in high-risk patients with aortic SVD.^6,7^ In these patients, ViV-TAVR has been associated with a high rate (95%) of procedural success and a mean 30-day mortality of 8%. Nevertheless, the Valve-in-Valve International Data (VIVID) Registry has identified several major concerns with this technique: coronary obstruction, valve malposition, and elevated residual gradients.^8,9^ Moreover, it is still unclear whether the elevated residual gradients after ViV procedures are comparable with those reported after redo surgical aortic valve replacement.^10,11^ Regarding post-procedural gradients, the type of transcatheter heart valve selected, as well as the implantation technique, are relevant factors, but they have not been investigated yet.^12,13^ Specifically, balloon post-dilation is a widely embraced strategy used to optimize transcatheter valve expansion, thereby positively affecting paravalvular leaks and mean gradients.

In this study, we aimed to analyse haemodynamic and clinical outcomes after ViV-TAVR and to compare the results according to the use of a balloon-expandable vs. self-expandable valve, with or without balloon post-dilation.

## Methods

### Study population

Between April 2008 and December 2020, 111 consecutive patients with symptomatic severe bioprosthetic SVD underwent ViV-TAVR at the Centro Cardiologico Monzino IRCCS in Milan, Italy. The decision to perform ViV-TAVR was made by a heart team based on established criteria.^6,7^ Patients with a predominant prosthetic-patient mismatch, thrombosis, endocarditis, or paravalvular regurgitation were excluded. We collected information about the size of the surgical prosthesis and the reason of valve failure. All ViV-TAVR procedures were performed under fluoroscopic guidance. In the majority of patients, transoesophageal echocardiography was used as an adjunct to fluoroscopy to provide an accurate evaluation of valve position and function, with careful assessment of the presence and severity of aortic regurgitation (AR). Patients were stratified according to percutaneous valve type and balloon post-dilation (balloon-expandable vs. self-expandable with and without post-dilation).

A comprehensive transthoracic echocardiography using commercially available equipment (iE33 or EPIQ, Philips Medical System, Andover, Massachusetts; Vivid-7, E9, or E95 GE Healthcare, Horten, Norway) was performed for all patients at baseline, discharge, and 6–12-months follow-up. All echocardiographic examinations were acquired by experienced echocardiographers. The transthoracic echocardiography was performed in multiple cross-sectional and off-axis views with careful attention to assessing the morphology and mobility of prosthetic valve leaflets, evaluating the integrity of the cusps, and detecting the presence of calcification. Left ventricular (LV) end-diastolic and end-systolic volumes and maximal left atrial volume were measured using the biplane Simpson’s method, and LV ejection fraction was derived. Doppler-derived parameters including peak velocity, mean pressure gradient, and velocity-time integral of the jet were assessed from the apical, right parasternal, right supraclavicular, and suprasternal positions. Prosthetic aortic valve area was derived from the continuity equation according to current guidelines.^14,15^ To avoid underestimation, the diameter of the LV outflow tract was measured in the mid-systole from the parasternal long-axis view using a zoomed mode, measuring inner-edge to inner-edge of the prosthetic valve. The LV outflow tract velocity-time integral was obtained using pulsed-wave Doppler by positioning the sample volume 5 mm below the prosthetic aortic leaflets. All echocardiographic measurements were obtained by averaging the values collected over three consecutive cardiac cycles in patients with sinus rhythm, and over five cardiac cycles for those with atrial fibrillation. In the presence of atrial fibrillation, care was taken to obtain measurements during less irregular heart rate, trying to match temporally similar beats for preventing unrepresentative results. To evaluate the presence and severity of prosthetic AR, a combination of the qualitative and semi-quantitative parameters was used according to current guidelines and to VARC-3 document.^14–16^

Clinical events were evaluated according to the Valve Academic Research Consortiun-3 criteria.^16^ The follow-up period was defined as the time between the procedure and the last documented contact with the patient alive or the time of documented death. All-cause mortality rate was reported during the 5-year follow-up period.

The Institutional Review Board and the Ethical Committee of our institution approved the study (CCM–PR182). For retrospective analysis of clinically acquired data anonymously handled, the Ethical Committee waived the need of patient written informed consent.

### Statistical analysis

Continuous data are presented as mean ± SD and categorical variables as numbers (percentages), as appropriate. Continuous variables were compared using the one-way analysis of variance test (for normally distributed variables) or the Kruskal–Wallis test (for non-normally distributed variables). Bonferroni *post hoc* analysis was performed to assess between-group differences in case of a significant difference in the overall 3-group comparison. Categorical variables were compared using the Pearson *χ*^2^ test or Fischer-exact test (if the expected cell count was <5) for categorical variables. ANOVA for repeated measures was used to evaluate changes in echocardiographic variables over time for the three groups. Mortality rates are presented as Kaplan–Meier curves, and the log-rank test was used for comparisons among groups. All *P* values <0.05 were considered statistically significant. Data analyses were conducted using SPSS version 27 (IBM SPSS Statistics, IBM Corporation, New York) and R version 4.1.2.

## Results

Successful ViV-TAVR was performed in 110 (99%) patients. Intraoperative mortality occurred in 1 patient who received a balloon-expandable valve due to rupture of the aortic valve annulus. The mechanism of valve failure was stenosis in 27 patients, regurgitation in 54 patients, and combined stenosis with regurgitation in 29 patients.

The balloon-expandable Edwards SAPIEN valve (Edwards Lifesciences, Irvine, California, USA) was used in 35 (32%, Group 1) patients, while a self-expandable valve (CoreValve, Medtronic, Minneapolis, Minnesota, USA and Portico, Abbott, Lake Bluff, Illinois, USA) was used in 76 cases. Among these cases, 39 (35%, Group 2) did not undergo balloon post-dilation, while 37 (33%, Group 3) did undergo balloon post-dilation.

Clinical and echocardiographic baseline characteristics of the entire population are presented in *Table 1*. The distribution of valve failure modes did not differ among the groups: Group 1 [stenosis, *n* = 14 (40%); regurgitation, *n* = 11 (41%); combined, *n* = 10 (29%)], Group 2 [stenosis, *n* = 18 (46%); regurgitation, *n* = 14 (36%); combined, *n* = 7 (18%)] and Group 3 [stenosis, *n* = 13 (35%); regurgitation, *n* = 16 (43%); combined, *n* = 8 (22%)]. There was no significant difference between the self-expandable and balloon-expandable valves in the rate of ViV-TAVR performed in small prosthesis [surgical valve size ≤21 mm: Group 1, 17 (51%); Group 2, 19 (53%); Group 3, 20 (57%); *P* = 0.886).

**Table 1:** Baseline clinical and echocardiographic characteristics of the overall population and comparison among groups

	All (*n* = 111)	Group 1 (*n* = 35)	Group 2 (*n* = 39)	Group 3 (*n* = 37)	*P* Value
**Clinical characteristics**					
Age, years	78 ± 8	77 ± 11	78 ± 7	79 ± 5	0.409
Female	57 (51%)	17 (49%)	19 (49%)	21 (56%)	0.723
Body Surface Area, kg/m^2^	1.76 ± 0.19	1.76 ± 0.19	1.75 ± 0.20	1.77 ± 0.20	0.938
Hypertension	90 (81%)	27 (77%)	31 (79%)	32 (86%)	0.570
Diabetes mellitus	27 (24%)	10 (29%)	11 (28%)	6 (16%)	0.371
Dyslipidaemia	71 (64%)	23 (66%)	20 (51%)	28 (76%)	0.083
Angina	16 (14%)	7 (20%)	6 (15%)	3 (8%)	0.349
Dyspnoea	110 (99%)	34 (97%)	39 (100%)	37 (100%)	0.334
Syncope	2 (2%)	2 (6%)	0 (0%)	0 (0%)	0.110
Chronic obstructive pulmonary disease	29 (26%)	12 (35%)	11 (28%)	6 (16%)	0.180
eGFR < 60 mL/min/1.73 m^2^	68 (61%)	20 (57%)	26 (67%)	22 (59%)	0.677
NYHA functional classes III–IV	86 (77%)	28 (80%)	33 (85%)	25 (68%)	0.187
Peripheral vascular disease	30 (27%)	10 (29%)	10 (25%)	10 (27%)	0.961
Coronary artery disease	40 (36%)	13 (37%)	15 (38%)	12 (32%)	0.849
Prior myocardial infarction	10 (9%)	4 (11%)	4 (10%)	2 (5%)	0.634
Prior percutaneous coronary intervention	21 (19%)	4 (11%)	9 (23%)	8 (22%)	0.388
Prior coronary artery bypass graft	27 (24%)	8 (23%)	10 (26%)	9 (24%)	0.962
Atrial flutter/fibrillation	24 (22%)	8 (23%)	9 (23%)	7 (19%)	0.887
**Echocardiographic characteristics**					
LVEDV index, mL/m^2^	78 ± 30	78 ± 31	79 ± 29	78 ± 32	0.989
LVESV index, mL/m^2^	36 ± 22	36 ± 21	38 ± 22	34 ± 22	0.750
LVEF, %	56 ± 11	56 ± 11	54 ± 13	60 ± 10	0.082
LV mass index, g/m^2^	146 ± 44	151 ± 37	146 ± 47	142 ± 46	0.824
LA volume index, mL/m^2^	59 ± 22	67 ± 32	55 ± 14	55 ± 16	0.054
AVA, cm^2^	0.99 ± 0.47	0.89 ± 0.3	1.07 ± 0.53	1.01 ± 0.52	0.282
AVA index, cm^2^/m^2^	0.56 ± 0.26	0.51 ± 0.18	0.61 ± 0.31	0.56 ± 0.27	0.241
Mean pressure gradient, mmHg	38 ± 16	41 ± 15	36 ± 16	37 ± 17	0.373
Peak pressure gradient, mmHg	63 ± 24	67 ± 22	60 ± 23	63 ± 26	0.393
PASP, mmHg	45 ± 13	45 ± 12	45 ± 13	46 ± 13	0.895
Mitral regurgitation ≥2	59 (53%)	20 (57%)	14 (36%)	22 (59%)	0.051
Tricuspid regurgitation ≥2	34 (31%)	15 (43%)	7 (18%)	12 (32%)	0.065
Mechanism of failure					0.697
Stenosis	45 (41%)	14 (40%)	18 (46%)	13 (35%)	
Regurgitation	41 (37%)	11 (31%)	14 (36%)	16 (43%)	
Combined	25 (22%)	10 (29%)	7 (18%)	8 (22%)	

AVA, aortic valve area; eGFR, estimated glomerular filtration rate; EDV, end-diastolic volume; EF, ejection fraction; ESV, end-systolic volume; LA, left atrial; LV, left ventricular; NF-HG, normal flow high gradient; NYHA, New York Heart Association; PASP, pulmonary artery systolic pressure.

Baseline peak and mean transprosthetic gradients, LV volumes, ejection fraction, and pulmonary artery systolic pressure were similar among groups. A significant improvement in all echocardiographic parameters was observed in all patients over time. Specifically, a significant reduction in post-procedural transprosthetic gradients was observed at discharge and 6–12 months follow-up compared with baseline in all groups (*Table 2*). The gradient reduction remained consistent over time, even in patients with small surgical valves. Immediately after ViV-TAVR procedure, the lowest mean transprosthetic gradient was observed in Group 3 (12 ± 7 mmHg) compared with Group 1 and Group 2 (20 ± 9 mmHg and 17 ± 8 mmHg, respectively, *P* < 0.001). This result proved to be reproducible even when considering small surgical valve in all three groups, despite having higher mean transprosthetic gradients compared with larger surgical valves. Furthermore, this result was confirmed at 6–12 months follow-up (*P* < 0.001; *Table 2*).

**Table 2:** Comparison of echocardiographic parameters at baseline, discharge, and 6–12 months follow-up after TAVR

Variables	Baseline	Discharge	6–12 Months	*P* Value
**Group 1 (*n*** **=** **35)**				
LVEDV index, mL/m^2^	78 ± 30	70 ± 26	62 ± 21*^,^**	0.002
LVESV index, mL/m^2^	37 ± 22	34 ± 20	26 ± 14*^,^**	<0.001
LVEF, %	56 ± 11	54 ± 10	61 ± 9*^,^**	<0.001
LV mass index, g/m^2^	155 ± 39	135 ± 44*	132 ± 50*	<0.001
LA volume index, mL/m^2^	65 ± 28	56 ± 14	52 ± 15	0.053
AVA, cm^2^	0.87 ± 0.30	1.42 ± 0.40*	1.37 ± 0.31*	<0.001
AVA index, cm^2^/m^2^	0.49 ± 0.18	0.8 ± 0.21*	0.76 ± 0.17*	<0.001
Mean pressure gradient, mmHg	41 ± 16	20 ± 9*	23 ± 10*	<0.001
Peak pressure gradient, mmHg	67 ± 24	34 ± 15*	39 ± 16*	<0.001
PASP, mmHg	42 ± 8	36 ± 7*	36 ± 9*	<0.001
Aortic valve regurgitation ≥2	21 (60%)	2 (7%)	2 (7%)	<0.001
Mitral regurgitation ≥2	18 (60%)	11 (37%)	8 (27%)*	0.008
Tricuspid regurgitation ≥2	12 (40%)	6 (20%)	2 (7%)*	0.003
**Group 2 (*n*** **=** **39)**				
LVEDV index, mL/m^2^	75 ± 27	66 ± 27*	60 ± 21*	<0.001
LVESV index, mL/m^2^	36 ± 22	31 ± 21	26 ± 17*	0.001
LVEF, %	55 ± 13	56 ± 11	58 ± 11	0.132
LV mass index, g/m^2^	144 ± 47	128 ± 39	122 ± 29*	0.014
LA volume index, mL/m^2^	54 ± 15	53 ± 16	53 ± 14	0.856
AVA, cm^2^	1.00 ± 0.53	1.62 ± 0.54*	1.59 ± 0.46*	<0.001
AVA index, cm^2^/m^2^	0.58 ± 0.31	0.92 ± 0.29*	0.9 ± 0.24*	<0.001
Mean pressure gradient, mmHg	38 ± 16	18 ± 9*	18 ± 11*	<0.001
Peak pressure gradient, mmHg	63 ± 23	32 ± 16*	32 ± 17*	<0.001
PASP, mmHg	44 ± 12	34 ± 9*	37 ± 10*	<0.001
Aortic valve regurgitation ≥2	21 (53%)	4 (13%)	4 (13%)	<0.001
Mitral regurgitation ≥2	11 (36%)	5 (16%)	5 (16%)	0.038
Tricuspid regurgitation ≥2	5 (16%)	2 (7%)	4 (13%)	0.247
**Group 3 (*n*** **=** **37)**				
LVEDV index, mL/m^2^	79 ± 33	63 ± 27*	61 ± 23*	<0.001
LVESV index, mL/m^2^	34 ± 23	30 ± 21*	26 ± 17*	<0.001
LVEF, %	60 ± 10	56 ± 10*	59 ± 9	0.011
LV mass index, g/m^2^	148 ± 48	124 ± 35*	116 ± 33*	<0.001
LA volume index, mL/m^2^	56 ± 18	49 ± 17	48 ± 18	0.043
AVA, cm^2^	0.98 ± 0.49	1.71 ± 0.43*	1.55 ± 0.48*	<0.001
AVA index, cm^2^/m^2^	0.54 ± 0.24	0.97 ± 0.25*	0.89 ± 0.28*	<0.001
Mean pressure gradient, mmHg	36 ± 16	12 ± 6*	13 ± 7*	<0.001
Peak pressure gradient, mmHg	62 ± 24	21 ± 10*	24 ± 11*	<0.001
PASP, mmHg	46 ± 13	36 ± 9*	39 ± 9*	<0.001
Aortic valve regurgitation ≥2	24 (64%)	2 (6%)	2 (6%)	<0.001
Mitral regurgitation ≥2	23 (68%)	16 (47%)*	9 (27%)*^,^**	<0.001
Tricuspid regurgitation ≥2	12 (35%)	10 (29%)	7 (21%)	0.150

^*^ *P* < 0.05 vs. Baseline.

^**^ *P* < 0.05 vs. Discharge.

AVA, aortic valve area; eGFR, estimated glomerular filtration rate; EDV, end-diastolic volume; EF, ejection fraction; ESV, end-systolic volume; LA, left atrial; LV, left ventricular; NF-HG, normal flow high gradient; NYHA, New York Heart Association; PASP, pulmonary artery systolic pressure.

No significant difference was found in paravalvular regurgitation rate among different SVD modes [stenosis, 2 (5%); regurgitation, 5 (14%); combined, 1 (5%); *P* = 0.325) at 6–12 months.

**Figure 1:**
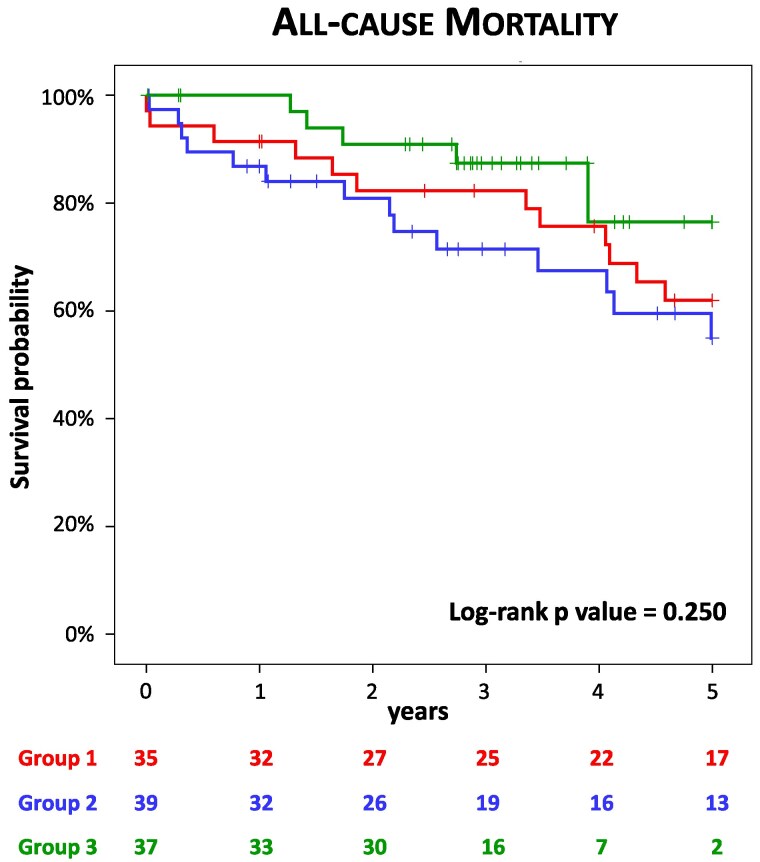
Kaplan–Meyer analysis. Kaplan–Meier curves demonstrating the event-free survival for all-cause mortality according to transcatheter valve type with or without balloon post-dilation (left panel) and mechanism of degeneration of the surgical valve (right panel).

Kaplan–Meier analysis demonstrated similar outcomes up to 5 years regardless of the transcatheter valve type with or without balloon post-dilation and regardless of the mechanism of degeneration of the surgical valve (*Figure 1*, *Table 3*). No difference in mortality rate was found between small and large surgical degenerated valves (*P* = 0.520).

**Table 3:** All-cause mortality according to transcatheter valve type with or without balloon post-dilation and mechanism of degeneration of the surgical valve

	Group 1 (*n* = 35)	Group 2 (*n* = 39)	Group 3 (*n* = 37)	*P* value
1-year	3 (9%)	5 (13%)	0 (0%)	0.090
3-year	6 (17%)	10 (26%)	4 (11%)	0.240
5-year	12 (34%)	14 (36%)	5 (14%)	0.056

	Stenosis (*n* = 45)	Regurgitation (*n* = 41)	Combined (*n* = 25)	*P* value
1-year	2 (4%)	4 (10%)	2 (8%)	0.711
3-year	7 (16%)	9 (22%)	4 (16%)	0.876
5-year	14 (31%)	11 (27%)	6 (24%)	0.801

**Figure 2:**
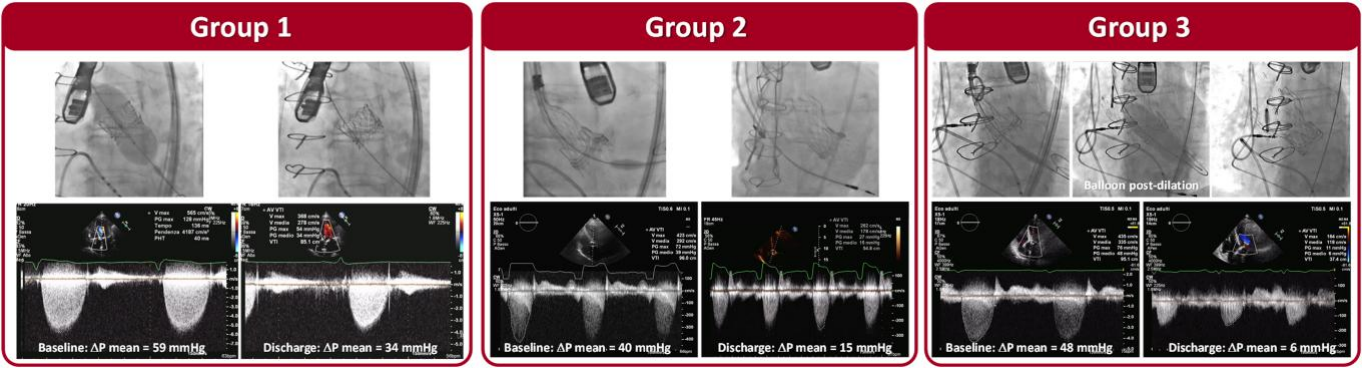
Representative cases. Fluoroscopy and continuous-wave Doppler tracings showing mean pressure gradient at baseline and discharge in a patient from Group 1 (Left Panel), Group 2 (Central Panel), and Group 3 (Right Panel).

*Figure 2* shows representative cases of fluoroscopy and transprosthetic gradients in Group 1, Group 2, and Group 3, respectively.

## Discussion

Until a few years ago, the best treatment of choice for prosthetic valve dysfunction has been redo surgery.^6,7^ However, patients with prosthetic aortic valve degeneration are increasingly older and have multiple comorbidities. As a result, they are not ideal candidates for redo surgery. Although ViV-TAVR may be an effective and less-invasive strategy for patients with failed surgical bioprostheses, suboptimal haemodynamic results might be a limitation of this procedure.^8–13^

This study includes a cohort of consecutive patients undergoing ViV-TAVR who were stratified into three groups: Group 1 receiving a balloon-expandable prosthesis, Group 2 receiving a self-expandable prosthesis without balloon post-dilation, and Group 3 receiving a self-expandable prosthesis with post-balloon dilatation. The main results of the study are: (i) significant improvement in all echocardiographic parameters in all groups both acutely and at follow-up; (ii) the lowest values of mean transprosthetic gradients immediately after implantation and at 6–12 months follow-up were obtained with the self-expandable prosthesis with balloon post-dilation; (iii) transcatheter valve type with or without balloon post-dilation and degeneration mode of the surgical valve were not associated with worse early and long-term all-cause mortality. However, there is a trend toward better outcomes in patients receiving a self-expandable prosthesis with balloon post-dilation.

The ViV-TAVR approach includes a highly heterogeneous group of procedures, applicable to various surgical valves with different degeneration modes.^17^ Accurate candidate assessment is crucial for optimal results, primarily based on knowledge of the failed bioprosthetic valve (model, size, structure, leaflet position, and failure mode). Another key factor is understanding the structural features and implantation techniques of transcatheter valves in relation to patient anatomy and type of surgical valve.^8,9^ Unlike previous studies,^9,18^ we observed a significant reduction in post-procedural gradients in all patients, regardless of prosthesis type. This may be influenced by improved knowledge of transcatheter valves features and implantation technique. Self-expandable valves with a supra-annular design minimize interference with the surgical valve annulus reducing transvalvular gradients, though deep deployment may lead to suboptimal haemodynamics. On the other hand, balloon-expandable valves, which are intra-annular devices, may be implanted in a higher position trying to be as ‘supra-annular’ as possible to achieve better haemodynamic.^19–21^ The significant reduction of post-procedural gradients observed in our study may have been due to the exclusion of cases with prevalent patient–prosthesis mismatch without severe bioprosthetic degeneration. Excluding patients with high transprosthetic gradients due to an effective orifice area of the surgical valve being too small in relation to body size in the absence of SVD (calcification/hypomobility of the leaflets or pannus) is essential for good haemodynamic results with ViV-TAVR. While prosthesis–patient mismatch can predispose to SVD,^22,23^ our findings suggest proper patient selection, ensuring that prosthesis–patient mismatch was not the primary dysfunction mechanism.

The absence of significant prosthetic regurgitation after ViV-TAVR is another relevant data of our study. The global registry of ViV-TAVR reported significant regurgitation in ≈5% of patients,^8^ primarily due to transcatheter valve malposition with paravalvular leak, sometimes mistaken as intravalvular regurgitation. The low rate of significant paravalvular regurgitation supports the good selection of our population, mainly due to the use of transaesophageal echocardiography during preoperative evaluation. This allowed for the exclusion of patients with both SVD and significant paravalvular leak, a condition that cannot be corrected with ViV-TAVR.

Despite the haemodynamic benefits of ViV-TAVR, some studies have shown that when small surgical valves are treated with this procedure, transvalvular gradients remain significantly increased during follow-up, even after an initial adequate reduction.^9,24^ In fact, in the PARTNER-2 study, surgical bioprosthetic valves <21 mm were exclued.^25^ Our study confirms sustained long-term gradient reduction, supporting the efficacy of the ViV-TAVR procedure regardless of the mechanism of SVD or surgical valve size, provided proper patient selection excludes prevalent patient–prosthesis mismatch. In addition, late valve thrombosis reported in ∼7% of cases may contribute to gradient elevation, though often asymptomatic.^26^ In this study, the lowest values of mean transprosthetic gradient were obtained with self-expandable prosthesis after balloon post-dilation, consistent with *ex vivo* bench test showing improved expansion after valve ballooning and improved residual gradients.^27^ However, high-pressure balloon inflation after ViV-TAVR may cause structural damage to the self-expanding valve frame or leaflets, resulting in severe acute regurgitation, or early and accelerated SVD. It is reassuring that we did not observe severe prosthesis regurgitation or early prosthesis degeneration up to 6–12 months follow-up in Group 3. Of course, operator experience and careful attention to balloon size and position might have played a key role in reducing the risk of these complications.

Another key finding of our study is the overall good survival across all three groups, with a trend toward better outcomes in patients receiving a self-expandable valve with balloon post-dilation, which correlates with lower transprosthetic gradients. As shown in previous studies,^18,19,28,29^ short-term survival in our cohort of patients with different transcatheter heart valves and procedure techniques was similar. In the PARTNER-2 registry,^30^ 3-year all-cause mortality was 32%, while in the CoreValve US Expanded Use Study it was 15%.^31^ Our data show an estimated mortality of 17% at 3 years, regardless of the prosthesis implanted and/or the implantation technique, which is in line with the literature. Furthermore, no differences in mortality were documented according to the underlying mechanism of dysfunction (stenosis, regurgitation, or combined). A recent study by Bleiziffer *et al*.^29^ reported lower survival rates in patients with failed bioprostheses having small diameters compared to those with large diameters. Indeed, a small surgical bioprostheses represents an important risk factor for mortality after ViV-TAVR. In our study, the proportion of patients with a labelled size of the surgical valve ≤21 mm was comparable among the three groups, and we found similar mortality rate in small and large degenerated surgical valves.

## Limitations

Several limitations should be acknowledged. First, this is a single-centre study analysing a limited number of patients, with the inherent structural bias of the study design. Second, VIV-TAVR is performed in a heterogeneous group of patients in terms of types and sizes of surgical and transcatheter valves. Third, the choice of transcatheter valve type (balloon-expandable vs. self-expandable valve) as well as the decision to perform post-dilation were determined by operator preference.

## Conclusions

In conclusion, when comparing the data from this study with the currently available literature, it is evident that the clinical and haemodynamic results of ViV-TAVR are satisfactory, and the procedure is safe and effective. Self-expandable valves with balloon post-dilation demonstrate lower post-procedural gradients, and thus this phenomenon is worthy of further investigation in a randomized controlled trial.

Several adverse events (elevated post-procedural gradients, persistent regurgitation, and thrombosis) have been described after ViV-TAVR, but they may be prevented with appropriate procedural planning, optimal prosthesis selection, increased operator experience, and novel transcatheter techniques such as ring fracture of the surgical bioprosthetic valve.

## Consent

For retrospective analysis of clinically acquired data anonymously handled, the Ethical Committee waived the need of patient written informed consent.

## Data Availability

Data are available upon reasonable request from the corresponding author.

## Lead author biography

Dr. Manuela Muratori is the Director of the Echocardiographic Laboratory within the Perioperative Cardiology and Cardiovascular Imaging Department led by Prof. Gianluca Pontone, at Centro Cardiologico Monzino IRCCS in Milan. She has published more than 140 articles in peer-reviewed international journals and has an H-index of 36 (Scopus). Her main field of research is echocardiography, specifically as it applies to valvular heart disease and valvular prostheses.
